# Penile Injuries in Immunocastrated and Entire Male Pigs of One Fattening Farm

**DOI:** 10.3390/ani7090071

**Published:** 2017-09-13

**Authors:** Simon Reiter, Susanne Zöls, Mathias Ritzmann, Volker Stefanski, Ulrike Weiler

**Affiliations:** 1 Clinic for Swine, Ludwig-Maximilians-University Munich, Sonnenstrasse 16, 85764 Oberschleissheim, Germany; s.reiter@med.vetmed.uni-muenchen.de (S.R.); s.zoels@lmu.de (S.Z.); schweineklinik@med.vetmed.uni-muenchen.de (M.R.); 2 Institute of Animal Science, Hohenheim University, Schloss Hohenheim 1, 70599 Stuttgart, Germany; volker.stefanski@uni-hohenheim.de

**Keywords:** immunocastrates (IC), Improvac^®^, entire male (EM), penile injuries

## Abstract

**Simple Summary:**

In the EU, stakeholders of the pork chain committed themselves to voluntarily end surgical castration of boars in Europe. Pork production with entire males (EM) and with immunocastrated boars (IC) are discussed as animal friendly alternatives to surgical castration. However, raising EM may cause new welfare problems due to sexual and aggressive behavior patterns, such as penile injuries. The incidence of this welfare problem with immunocastrated pigs has not been described so far. Thus, it was the aim of this study to compare frequency and severity of penile injuries in EM and IC systematically. Incidence and size of penile injuries (wounds, scars, hematomas) were evaluated in 192 IC and 215 EM from one farm after slaughter. Scars were observed in 71.2% EM and 44.8% IC; whereas wounds were obvious only in 17.2% EM and 8.3% IC. Thus, it is concluded that immunocastration reduces the frequency and severity of penile injuries in IC when compared to EM of same age and weight.

**Abstract:**

Penile injuries in boars have been discussed as a relevant welfare problem in pork production with entire males (EM). The incidence of penile injuries with immunocastrated boars has not been described so far. Thus, it was the aim of this study to systematically compare frequency and severity of penile injuries in EM and IC. Incidence and size of penile injuries (wounds, scars, hematomas) were evaluated in 192 IC and 215 EM from one farm after slaughter (120 kg live weight; four batches (BA) in at least weekly intervals over five weeks). 75.8% EM and 48.4% IC showed injuries at the pars libra of the penis. Scars were observed in 71.2% EM and 44.8% IC. Scars/animal were significantly influenced by treatment (IC vs. EM), B and treatment x B and increased with age in EM (BA1: 2.61 ± 3.05; BA4: 3.59 ± 3.47), but not in IC (BA1: 2.00 ± 3.02; BA4: 1.22 ± 1.91). Wounds were obvious in 17.2% EM and 8.3% IC. Wounds/animal were only influenced significantly by treatment and were lower in IC than in EM. Thus, it is concluded that immunocastration reduces the frequency and severity of penile injuries in IC when compared to EM of same age and weight.

## 1. Introduction

In the EU, surgical castration of male piglets without pain relief is nowadays unacceptable. Stakeholders of the pork chain committed themselves to voluntarily end surgical castration of boars in Europe by 1st of January 2018. Some countries have already prohibited surgical castration of boars without pain relief by law. The amendment of the Protection Animals Act in Germany from 13th of July 2013 bans surgical castration of male piglets without pain release from 1st of January 2019. As a consequence, alternatives need to be evaluated. Besides surgical castration with anesthesia and analgesia, pork production with entire males (EM) and with immunocastrated boars (IC) have been discussed as animal friendly alternatives to piglet castration without pain relief [1].

Nevertheless, the use of EM for pork production in Germany is still limited because of meat quality problems caused by androstenone and skatole [2]. Additionally, behavioral problems increase due to aggressive behavior of EM in order to establish social ranking [3]. Furthermore, EM shows a pronounced increase in sexual behavior along the fattening period, e.g., a threefold higher rate of mountings compared to gilts. In EM sexual oriented mounting is characterized by prolonged duration and pelvic thrusts [4,5]. This mounting activity may cause welfare problems due to lameness and other skeletal problems in both the mounting and the mounted animal [5]. In addition, sexually oriented mounting may also lead to penile injuries due to penis biting [6]. 

In juvenile boars or barrows, the penile frenulum represents the connection between the preputial sheet and the penis and prevents the extrusion of the penis [7]. Through increasing pelvic thrusting during the pubertal development, the penile frenulum is disrupted and the boars acquire the ability to extrude the penis completely [7]. The extended penis of a mounting boar may stimulate other pen mates to bite and hereby injure the penis. In recent publications [6,8], a high incidence of penile injuries has been described for various populations of EM whereas, in surgical castrates, this problem was not detectable. It was concluded that penis biting is highly relevant for welfare problems in boars. 

Immunocastration is discussed as an alternative to surgical castration and is performed by vaccinating boars twice with an antigen that stimulates the production of anti-GnRH-antibodies. The two vaccinations are carried out with an interval of at least four weeks. The second vaccination should be given four to six weeks before slaughter (Improvac^®^, Zoetis Deutschland GmbH, Berlin, Germany). Interrupting the endocrine cascade of the hypothalamic-pituitary-axis with anti-GnRH-antibodies has the consequence that the synthesis of testicular steroids in Leydig cells ceases and returns to prepubertal levels [9]. Thus, one week after successful vaccinations, low testosterone and androstenone levels are measurable in vaccinated boars [10,11]. Between 10 and 14 days after the second vaccination, the hormone dependent aggressive and sexual behavior of boars starts to decrease [12,13]. However, consequences of IC for the incidence of penile injuries have not been described so far. Thus, it was the aim of the present study to systematically compare the frequency and severity of penile injuries in IC versus EM.

## 2. Materials and Methods

The aim of the present study was to quantify the incidence and severity of penile injuries in GnRH vaccinated IC and to compare them to EM raised under the same conditions. To allow a comparison with a previous study [6] a similar evaluation scheme was applied.

### 2.1. Animals and Sampling

In total, 407 animals were raised from an average weight of 30 kg (11 weeks old) to 120 kg in a commercial fattening farm in the northern part of Germany, which had been selected for the study due to a known experience in rearing EM. All animals from the trial derived from one farrowing group (Danzucht x Du) were transported to the fattening farm with 11 weeks of age and an average weight of 30 kg. Animals were raised in groups of 15 animals in fully slatted pens with sensor controlled liquid feeding and water was provided ad libitum. The animals were assigned to the two treatment groups EM and IC in the fattening unit. The IC (*n* = 192) were immunized twice (first and sixth week of fattening, corresponding to an age of 12 and 17 weeks) with anti-GnRH vaccine (2 mL Improvac^®^, Zoetis Deutschland GmbH, Berlin, Germany) and EM (*n* = 215) remained unvaccinated. The live weight at slaughter was 120–125 kg. The length of the finishing period until slaughtering ranged between 12 weeks (BA1) and 16 weeks (BA4), with 13 weeks (BA2) and 15 weeks (BA3) respectively. At the slaughter line, the genital tract (penis, covered by the preputial sheet) was excised during evisceration and further evaluated as described below in detail [6].

### 2.2. Evaluation of the Samples

**Figure 1:**
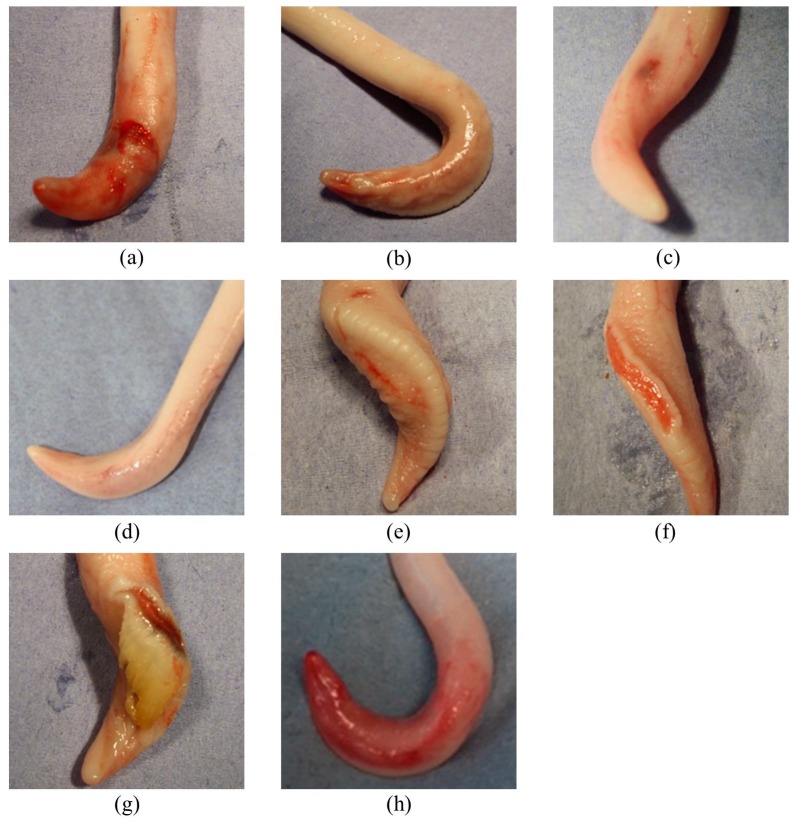
Pars libra penis with (**a**) wounds; (**b**) multiple scars; (**c**) hematoma; (**d**) no injuries; (**e**) slightly hypertrophic ridge; (**f**) slightly hypertrophic ridge with abrasions; (**g**) ridge with hyperkeratosis; (**h**) abrasion of the glans penis.

Preparation and evaluation of the samples were carried out according to earlier studies [6]. To evaluate the specimen, the penis was carefully pushed in a caudal direction within the preputial sheet, so the preputial sheet could be dissected without damaging the glans penis or the pars libra [6]. After removing the preputium, the pars libra penis was evaluated for different types of lesions: wounds, scars, hematomas, changes of the ridge (Figure 1a–g). In addition, the size of wounds and scars was recorded for each sample according to a size-score from 0.1–0.3 cm, >0.3–0.6 cm, >0.6–1 cm, >1 cm. Injuries >1 cm, with suppuration or losses of a part of penis were classified as ‘severe injuries’ [6]. The ridge was also classified in ‘physiological’, ‘slightly hypertrophic’ (Figure 1e), and ‘slightly hypertrophic with abrasions’ (Figure 1f). The total number of injuries includes scars, wounds, and hematomas. Moreover, other particularities that differ from the physiological anatomy of the boar penis were detected as ridge with hyperkeratosis (Figure 1g) or abrasions of the glans penis, which indicate sexual activity (Figure 1h).

### 2.3. Statistical Analysis

All statistical analyses were performed in IBM SPSS Statistics Program (Version 23). Associations of categorical variables/percent values with group membership were analyzed using the chi-square test. The relationship between number of wounds and number of scars was analyzed by calculating the coefficient of Spearman’s rank-correlation. As the total number of injuries per animal was not normally distributed, the nonparametric Mann–Whitney test was used for analysis of differences between groups and batches. A Poisson regression analysis (GLM) was carried out to evaluate the effects of batch and group as well as their interaction on the number of scars and wounds, respectively, and the respective effects were described by calculating the distribution via Poisson regression.

## 3. Results

### 3.1. Influence of Treatment and Age on the Number of Scars and Wounds

The Poisson regression analysis revealed a significant effect (each *p* < 0.001) of group BA and group x BA in case of scars. For the number of wounds only the group had a significant effect. The quantification is further shown in Table 1.

As shown in Table 1 (left side), the number of scars in EM was in total about 1.3-fold higher compared to IC. In EM the number of scars continuously increased from BA1 to BA4 up to 2.26-fold more scars (*p* < 0.001) compared to the reference category BA1. In IC, the mean number of scars was estimated to be reduced to the about 0.6-fold of that of BA1 (reference category) in all following batches. These findings confirm the results from Table 2. Regarding the mean number of wounds (Table 1, right side), only the factor group was significant (*p* < 0.01) with a 4.18-fold mean number of wounds in EM animals compared to IC animals. The type and size of wounds are further analyzed below. 

### 3.2. Incidence of Hyperkeratosis and Hypertrophia of the Ridge

In total, samples of 215 EM and 192 IC were evaluated. Hyperkeratosis on the ridge was obvious in 12.1% of EM and 5.2% of IC samples. A slightly hypertrophic ridge was observed in 27.9% of the samples from EM and 11.6% showed hypertrophic ridges with abrasions. In contrast, 19.3% of IC had slightly hypertrophic ridges and 7.3% of IC had slightly hypertrophic ridges with abrasions (*p* = 0.008). Five animals showed abrasions at the top of the penis (EM: *n* = 2; IC: *n* = 3). 

### 3.3. Incidence and Size of Penile Injuries

The incidence and severity of penile injuries per group and batch are summarized in Table 2 and Table 3. Overall, 75.8% of EM and 48.4% of IC showed signs of penile injuries (scars, wounds, and hematomas) (*p* < 0.001). This difference was even more pronounced when evaluating the number of samples with scars (EM = 71.2% vs. IC = 44.8% (*p* < 0.001)) than with wounds (EM = 17.2% vs. IC = 8.3% (*p* < 0.001)). The total number of injuries (scars, wounds, and hematomas) differed significantly between EM with 3.4 ± 3.4 injuries and IC with 1.7 ± 2.5 injuries (*p* < 0.001). In IC the number of scars correlates with the number of wounds (*r* = 0.172; *p* = 0.017) in contrast to EM group. 27 animals were classified to have penile hematomas (EM: *n* = 20.7%; IC: *n* = 23.0%).

In Table 2, the numbers of scars and wounds per group and batch are displayed. The number of scars per animal in EM increased from 2.61 ± 3.05 (BA1) to 3.59 ± 3.47 (BA4), in contrast to IC the number of scars decreased and remained on a lower level (BA1: 2.00 ± 3.02 vs. BA4: 1.22 ± 1.91). The number of wounds was lower in IC compared to EM through all batches.

According to size distribution of penile injuries (Table 3), EM showed generally more injuries in every score class, and the number of injuries in the score classes decreased continuously from the lowest (EM = 67.9%; IC = 43.2%) to the highest score class, which comprises severe injuries (EM = 9.3%; IC = 2.6%). 

## 4. Discussion

To our knowledge, this is the first study that evaluates and quantifies the incidence of penile injuries of GnRH vaccinated boars. It has been described already by others that EM display more sexual mounting and longer mounts than gilts [4]. Probably, this frequent mounting behavior explains the high incidence of penile injuries in EM in our study, as the extrusion of the penis during sexually oriented mounting is prerequisite for penis biting by pen mates. Penile injuries in boars have been discussed as a relevant welfare problem in EM even if scientific studies on this topic are scanty. In a previous publication from our group, it had been demonstrated that penile injuries are not a sporadic phenomenon, but occur in intensive production systems (64.0–94.9%) as well as in wild living boars (40%) [6].

In the present study IC had significantly less penile scars and fresh wounds than EM. This might be a consequence of the decreasing sexual behavior in IC after the second vaccination [12,13] due to the increasing level of GnRH antibodies and the concomitant decrease of testosterone synthesis by Leydig cells [10]. The suppressive effect of GnRH vaccination, especially on aggressive and sexual behavior, may be observed up to 22 weeks after the second vaccination in accordance to some investigations [11,14], whereas in another study that monitored Leydig cell function after the second vaccination by measuring testicular steroids in blood, the resumption of steroidogenesis differed extremely between individuals, and ranged between 10 and 24 weeks [15]. In comparison to results of surgical castrates, which are not able to extrude the penis, IC had more penile injuries due to the male behavior before the second vaccination [6]. 

In EM, the number of scars increased with age, as from batch one to batch four an almost linear increase of 0.25 from BA1 to BA4 could be observed. In contrast, IC showed no increasing tendency in the number of scars with age. An explanation for the continuous increase in the number of scars in EM might be the increasing level of sexual activity along the fattening period as described by others [8]. However, contradictory results have also been published [13,16], describing decreasing sexual activity with increasing weight. It has to be kept in mind, however, that aggressive and sexual behavior may be further modified by the management of the groups such as removing pen mates from a fattening group, which may lead to an increase in agonistic behavior in the remaining group two-fold during the following days [17]. EM and IC were selected according to weight for the BA and removed from pens in our study.

Irrespectively of the effects in EM, the results clearly show that GnRH vaccination of boars leads to a lower frequency of penile injuries and a lower severity of the injuries than in EM. For further studies, it may be suggested that the regular evaluation of penile injuries may provide an excellent indicator for sexual activity and mounting behavior in EM pork production.

## 5. Conclusions

The results show that immunocastration reduces the frequency and severity of penile injuries in IC when compared to EM raised under the same conditions.

## Figures and Tables

**Table 1:** Probability distribution (Poisson). Left side dependent variable: number of scars; right side dependent variable: number of wounds.

Parameter	Number of Scars per Animal			Number of Wounds per Animal		
	B	*p* Value	exp(B)	B	*p* Value	exp(B)
(constant term)	0.693	0.000	2.000	−2.197	0.000	0.111
[group = EM]	0.265	0.048	1.304	1.430	0.003	4.179
[group = IC]	*ref. cat.*		1	*ref. cat.*		1
[BA4]	−0.496	0.022	0.609	−0.938	0.392	0.391
[BA3]	−0.499	0.004	0.607	−0.847	0.311	0.429
[BA2]	−0.409	0.004	0.665	−0.735	0.144	2.085
[BA1]	*ref. cat.*		1	*ref. cat.*		1
[group = EM] * [BA4]	0.817	0.001	2.264	0.808	0.484	2.242
[group = EM] * [BA3]	0.738	0.000	2.092	0.206	0.821	1.228
[group = EM] * [BA2]	0.465	0.009	1.592	−0.823	0.145	0.439

*n* = 407. model: constant term, group, batch, group*batch. Regression coefficient (B); exponentiated regression coefficient (exp(B)); ref. cat. = reference category.

**Table 2:** Number of scars and number of wounds per animal (mean ± SD), percentage (%) of animals with injuries and with severe injuries of EM and IC per batch (BA) are given.

Group	BA	*n*	Number of Scars/Animal	Number of Wounds/Animal	% Animals with Injuries	% Animals with Severe Injuries
IC	1	45	2.00 ± 3.02	0.11 ± 0.53	48.89	2.22
	2	82	1.33 ± 2.35	0.23 ± 0.65	48.78	4.88
	3	42	1.21 ± 1.66	0.05 ± 0.31	52.38	0.00
	4	23	1.22 ± 1.91	0.04 ± 0.21	39.13	0.00
	Total	192	1.45 ± 2.35	0.14 ± 0.53	48.44	2.60
EM	1	56	2.61 ± 3.05	0.46 ± 1.37	73.21	14.29
	2	87	2.76 ± 3.13	0.43 ± 1.12	70.11	4.60
	3	45	3.31 ± 2.58	0.24 ± 0.68	88.89	4.44
	4	27	3.59 ± 3.47	0.41 ± 1.08	77.78	22.22
	Total	215	2.94 ± 3.05	0.40 ± 1.11	75.81	9.30
Total		407	2.24 ± 2.84	0.28 ± 0.89	62.90	6.14

**Table 3:** Size distribution of injuries in % of animals of IC and EM and significance of difference between IC and EM (chi-square test).

Group	*n*	Injury Size Class			
		<0.3 cm	0.3–0.6 cm	0.6–1.0 cm	>1.0 cm
IC	192	43.2%	12.0%	3.6%	2.6%
EM	215	67.9%	19.5%	6.5%	9.3%
Total	407	56.3%	16.0%	5.2%	6.1%
*p*		<0.001	<0.05	ns	<0.01
